# Genetic investigation of a Tunisian family with Lynch syndrome: a case report

**DOI:** 10.3389/fonc.2025.1695240

**Published:** 2026-01-14

**Authors:** Rania Abdelmaksoud-Dammak, Nihel Ammous-Boukhris, Souhir Guidara, Slim Charfi, Ameni Feki, Manel Guirat, Rahma Daoud, Hassen Kamoun, Tahya Sellami-Boudawara, Afef Khanfir, Raja Mokdad-Gargouri

**Affiliations:** 1 Center of Biotechnology of Sfax, Laboratory of Eukaryotes Molecular Biotechnology. University of Sfax, Sfax, Tunisia; 2 Department of Human Genetics, Hedi Chaker Hospital, University of Sfax, Sfax, Tunisia; 3 Department of Anatomo-pathology, Habib Bourguiba Hospital, University of Sfax, Sfax, Tunisia; 4 Department of Medical Oncology, Habib Bourguiba Hospital, University of Sfax, Sfax, Tunisia; 5 Department of Surgery, Habib Bourguiba Hospital, University of Sfax, Sfax, Tunisia

**Keywords:** colorectal cancer, Lynch syndrome, MMR genes, pathogenic variant, targeted-NGS

## Abstract

**Purpose:**

This study aimed to characterize the clinical and molecular features of a Tunisian family suspected of Lynch syndrome (LS) and identify the segregating pathogenic variant(s).

**Methods:**

A three-generation consanguineous family from the south of Tunisia with six members was recruited. Clinical diagnosis of LS was suspected according to the criteria of Amsterdam. A comprehensive molecular analysis was conducted, including immunohistochemical staining for mismatch repair (MMR) proteins, microsatellite instability (MSI) testing, targeted next-generation sequencing (NGS), and confirmatory Sanger sequencing. Iterative Threading ASSEmbly Refinement (I-TASSER) was used to analyze changes in the functional domains of mutant proteins.

**Results:**

Five members of the family developed cancer before the age of 45, including four cases of colorectal cancer and one case of glioblastoma. Immunohistochemical analysis of the proband showed complete loss of MSH2 and MSH6 protein expression, consistent with a high MSI (MSI-H) phenotype. Germline testing identified a pathogenic frameshift variant in MSH2 (NM_000251: c.687delA, p.Ala230LeuTer16) in the proband, her father, and two of her brothers, whereas her healthy sister did not carry the variant. An additional germline pathogenic variant in MUTYH (NM_001048171: c.1143_1144dupG, p.Glu382GlyTer43) was detected only in the proband’s father and one of her brothers and was absent in the proband. Moreover, the proband later developed an extracolonic malignancy, a right ovarian tumor. NGS analysis of the tumor tissue revealed a pathogenic BRCA2 variant (c.1813delA, p.Ile605TyrTer9), which provides a potential target for personalized therapy. This case report highlights the co-segregation of a rare pathogenic MSH2 variant in a Tunisian family and underscores its clinical implications for improving the management and surveillance of patients with Lynch syndrome.

## Background

Lynch syndrome (LS) is an autosomal dominant hereditary cancer predisposition syndrome caused by germline pathogenic variants in DNA mismatch repair (MMR) genes (1, 2). LS individuals are at markedly increased risk for several malignancies, especially colorectal, endometrial, and ovarian cancers (3, 4). The lifetime cancer risk in individuals with LS varies according to the MMR gene involved, with the cumulative lifetime risk of colorectal cancer (CRC) estimated to be as high as 52.2% in women and 68.7% in men (5). Approximately 15% of CRC cases demonstrate a deficiency in MMR, resulting in microsatellite instability (MSI) and the absence of MMR protein expression (6, 7). MMR genes such as MLH1, MSH2, MSH6, and PMS2 play crucial functions in maintaining genomic stability (8). The incidence of CRC varies according to the altered MMR gene, and higher cumulative incidence rates are reported in individuals carrying mutations in *MLH1* (36%–52%) and *MSH2* (30%–50%) compared to those with mutations in *MSH6* (10%–17%) and *PMS2* (3%–11%) genes (9).

Diagnosis of LS typically relies on a combination of immunohistochemistry (IHC) to assess MMR protein expression and microsatellite instability testing (10). The advent of next-generation sequencing (NGS) has further improved the detection of causal germline variants, enabling more accurate diagnosis and personalized management for patients and their at-risk relatives (11).

Despite these diagnostic advances, data on LS in North African populations, particularly in Tunisia, remain scarce. The spectrum of MMR gene variants, their clinical significance, and patterns of familial co-segregation are still poorly defined, limiting the development of tailored surveillance and preventive strategies for Tunisian patients.

To address this gap, the present study characterizes the clinical, pathological, and genetic features of a Tunisian family suspected of LS, in which five members developed early-onset colorectal cancer, glioblastoma, or ovarian cancer. Through IHC, MSI testing, and NGS analysis, we report a germline MSH2 frameshift mutation, detected either alone or in combination with a pathogenic MUTYH variant, and evaluate its co-segregation with disease within the family.

## Methods and materials

### Patients

We report the case of a Tunisian family with five members exhibiting clinical features of LS according to Amsterdam II criteria. The medical records of the subjects were consulted, and the clinicopathological features were noted, such as sex, age of onset, age of death, location of colorectal tumors, and extracolonic tumors. This study was approved by the Ethics Committee of the Faculty of Medicine of Sfax-Tunisia. Written informed consent for publication of clinical details and genetic results was obtained from all participating family members.

### Targeted next-generation sequencing

Genomic DNA was isolated from the peripheral blood sample of patient II-5 diagnosed with CRC using the QIAamp DNA Blood Mini Kit (Qiagen, Hilden, Germany), following the manufacturer’s instructions. DNA was quantified using the Qubit 3.0 Fluorometric Quantitation (Thermo Fisher Scientific, Waltham, Massachusetts, USA). An aliquot of 100 ng of genomic DNA was used to prepare the library for targeted NGS using a panel including genes usually associated with CRC (*APC*, *BMPR1A*, *CDH1*, *EPCAM*, *MSH2*, *MSH6*, *MLH1*, *MUTYH*, *PMS2*, *POLD1*, *POLE*, *PTEN*, *SMAD4*, and *STK11*).

For BRCA testing, genomic DNA was extracted from ovarian biopsy using the QIAamp FFPE Tissue Kit (Qiagen). A DNA library for the detection of short structural variants [single-nucleotide variants (SNVs) and insertions/deletions (INDELs)] was generated using the OncoReveal BRCA1/2+CNV Panel (Pillar) as recommended by the manufacturer.

The libraries were quantified using the Qubit^®^ dsDNA HS Assay Kit (Life Technologies, Carlsbad, CA, USA) and then pooled and prepared for sequencing using the MiSeq Reagent Kit v3 (300 cycles) (Illumina, San Diego, CA, USA). This procedure generated paired-end reads with a 151-bp read length. Reads were trimmed to remove low-quality sequences and then aligned to the human reference genome (GRCh37/hg19) using the Burrows-Wheeler Alignment (BWA) package. Sequences from the National Center for Biotechnology Information (NCBI) database (http://www.ncbi.nlm.nih.gov) were used as the reference.

### Sanger sequencing

Sanger sequencing was used to confirm pathogenic variants identified by targeted NGS and to perform familial segregation analysis. Forward and reverse primers were designed using the Primer 3.0 software to amplify the fragments covering the variant region in exon 4 of *MSH2* (forward 5′-GCACATTGTATAAACA TTTAATGTAGGTGAATCTG-3′ and reverse 5′-ATATGACAGAAATATCCTTC-3′) and exon 13 of *MUTYH* (forward 5′-GCCCCTAAAGC CCTCTTGG-3′ and reverse 5′-GTGGATATAGCCTCAAAAGCCA-3′). PCR products were purified and labeled using the BigDye Terminator V3.1 Cycle Sequencing Kit and sequenced on the SeqStudio (Applied Biosystems, Foster City, CA, USA). Sequence analysis was performed using the BioEdit software.

### Immunohistochemistry of MMR proteins

Tissue specimens from the proband (II-3) were collected from a biopsy of paraffin-embedded samples for IHC analysis. Briefly, sections of formalin-fixed paraffin-embedded (FFPE) tissue were mounted on poly-l-lysine-coated slides, and non-specific proteins were blocked using the blocking solution. Then, the sections were incubated with primary antibodies for MSH6, MSH2, MLH1, and PMS2 proteins (Novocastra primary antibodies, Leica Biosystems, Heidelberger, Germany) and then visualized using Novolink Max Polymer Detection Systems (Leica Biosystems, Heidelberger, Germany). Slides were finally assessed and scored by the pathologists according to the extent of immunostaining.

### Microsatellite instability analysis

DNA samples extracted from FFPE colorectal tumor tissue and matched non-tumorous mucosa were used for MSI testing. Six monomorphic microsatellite markers (NR21, NR24, NR27, BAT25, BAT26, and HSP110) were PCR-amplified, and the resulting amplicons were analyzed using the ABI SeqStudio Genetic Analyzer (Applied Biosystems). MSI status was determined according to National Cancer Institute (NCI) guidelines by comparing allelic profiles between tumor and germline DNA.

### Three-dimensional structure

The three-dimensional (3D) structure of wild-type and mutated MSH2 proteins was analyzed and displayed using I-TASSER.

## Results

### Clinical features

**Figure 1:**
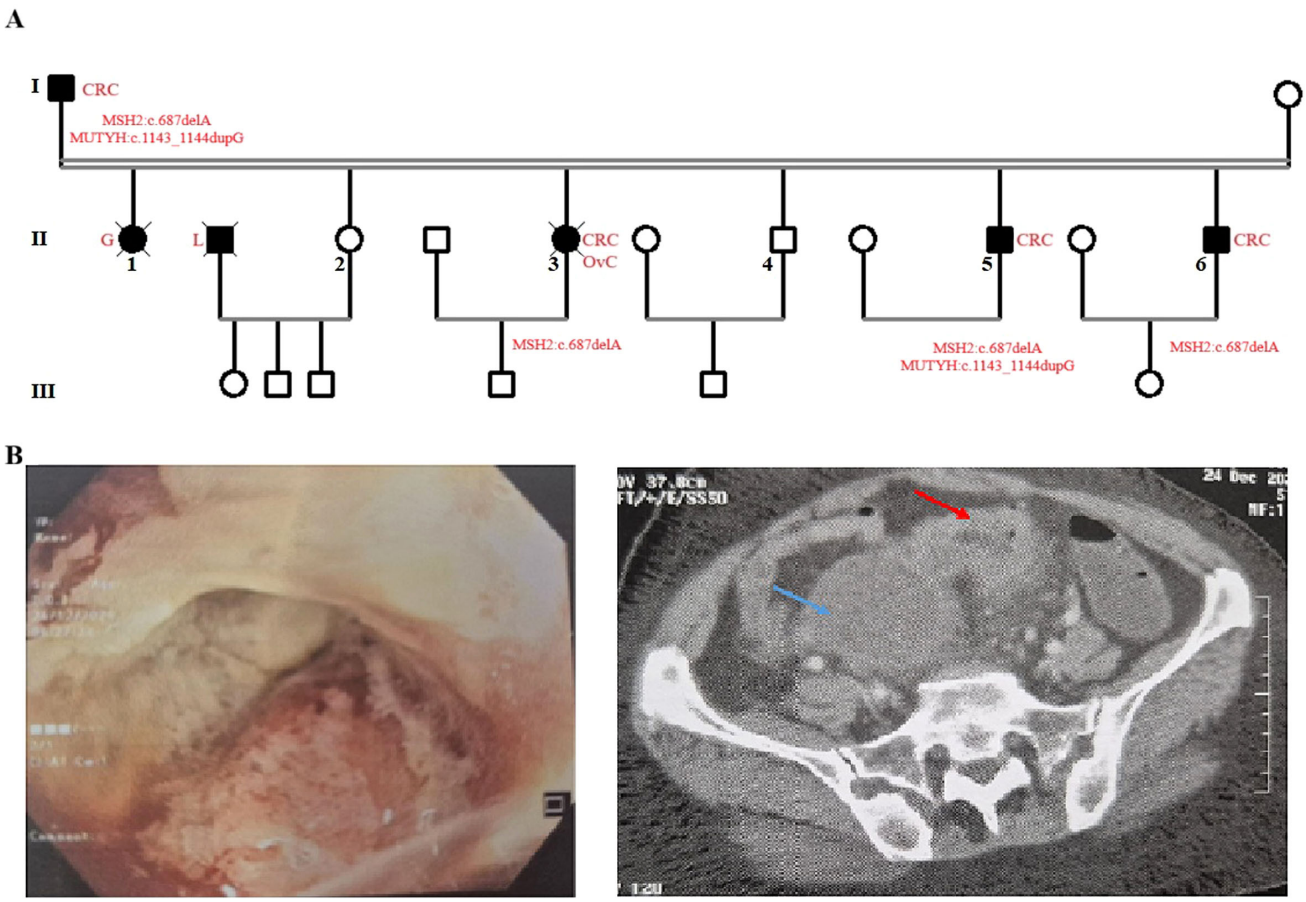
Pedigree of the Lynch syndrome (LS) family. Male individuals are indicated by squares and female individuals by circles, and cancer history in the family is indicated in red **(A)**. Colonoscopy and CT of the proband. Blue arrow indicates ovarian mass, and red arrow shows irregular thickening of the colon **(B)**.

We report a Tunisian family with five members diagnosed with primary cancers (**Figure 1A**). The proband (II-3) developed two cancers (CRC and ovarian cancer); therefore, Lynch syndrome was suspected based on the Amsterdam II criteria. Here, we described the detailed clinical manifestations of all patients.

Patient I-1: He was diagnosed with cancer when he was 40. He underwent radical resection for sigmoid colon cancer and was treated with chemotherapy and radiotherapy. Later on, he was re-diagnosed with colon cancer and underwent surgery again. The patient is 80 years old and has a tumor-free survival.

Patient II-1: She was diagnosed with glioblastoma at the age of 40, received chemotherapy without surgery, and died a few months later.

Patient II-3: She is the proband who was diagnosed at 45 with cecum cancer (**Figure 1B**). In March 2023, she underwent surgery, and the pathological examination concluded moderately differentiated adenocarcinoma. In February 2024, the patient was treated by laparotomy, revealing intraperitoneal carcinosis with a 3-cm latero-uterine mass encasing the sigmoid colon. Pathological diagnosis indicated grade III ovarian carcinoma, and she underwent first-line chemotherapy (Taxol-Carbo), which was completed on 20 August 24. CT evaluation showed a largely necrotic latero-uterine mass invading the ureter, sigmoid, uterus, and bladder (**Figure 1B**). On 11 April 2025, the patient underwent resection of the sigmoid, uterus, and bladder, and the pathological diagnosis revealed pelvic infiltration by a poorly differentiated carcinoma, probably of gynecological origin. She received chemotherapy (carboplatin and gemcitabine–avastin). She died at the age of 47.

Patient II-5: He was diagnosed with colon cancer at the age of 44. He underwent radical resection.

Patient II-6: He was diagnosed with colon cancer at the age of 39. He underwent radical resection.

No additional treatment information could be retrieved from the records.

### Immunohistochemical analysis and MSI testing

**Figure 2:**
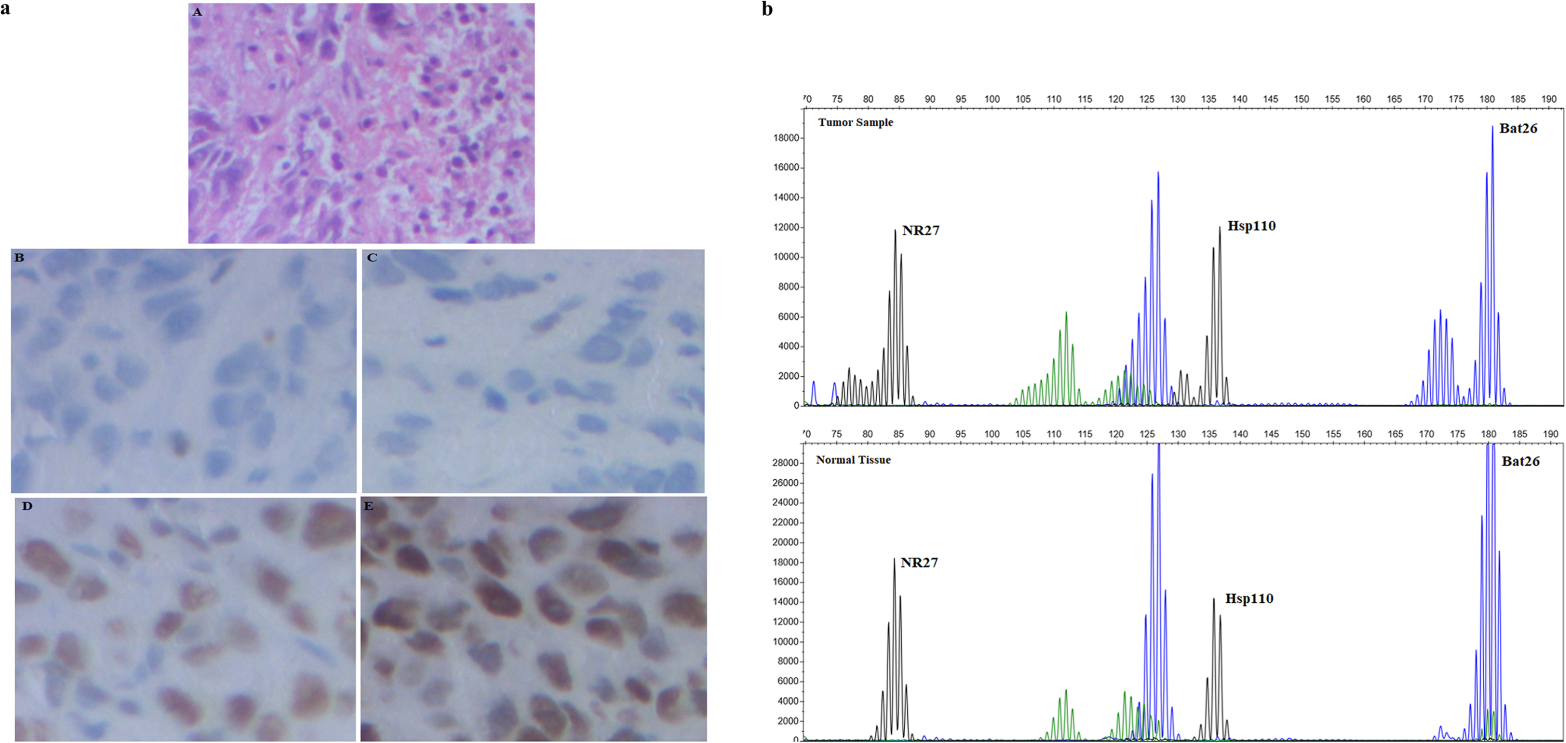
**(a)** Immunohistochemistry showing the expression of mismatch repair (MMR) proteins: (A) hematoxylin and eosin (H&E). Immunostaining was negative for MSH2 (B) and MSH6 (C) and positive for MLH1 (D) and PMS2 (E). **(b)** PCR-based microsatellite instability (MSI) testing showing microsatellite instability in one of six mononucleotide markers for tumor tissues and non-tumoral tissues.

IHC analysis of the tumor of the proband (II-3) was conducted to analyze the expression of MLH1, MSH2, MSH6, and PMS2 proteins. The result showed negative staining for MSH2 and MSH6 (**Figure 2a**), whereas MLH1 and PMS2 displayed a positive signal (**Figure 2a**), suggesting the deficiency in the MMR pathway in the proband.

The MSI test was performed to evaluate the deficiency of MMR function. Tumor and normal tissues from the proband (II-3) were subjected to MSI testing. The results showed that two of five markers were unstable, indicating MSI-high (MSI-H) phenotype (**Figure 2b**).

### Identification of genetic variants

**Figure 3:**
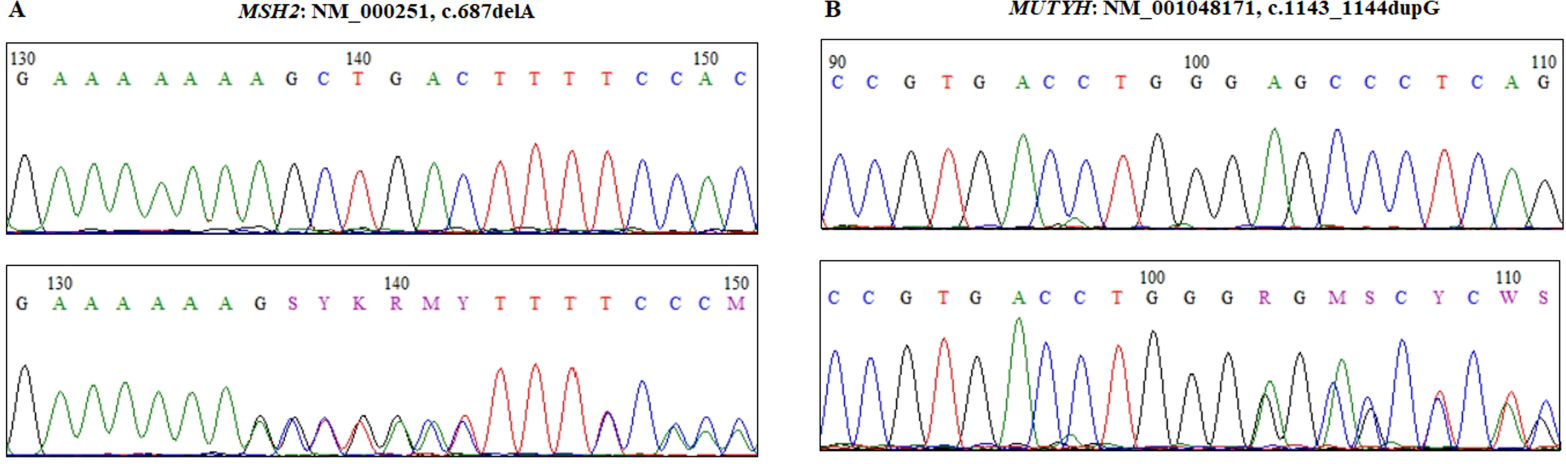
Sanger sequencing showing germinal variants in *MSH2***(A)** and *MUTYH***(B)** genes at heterozygous state. Upper chromatograms show the wild-type sequences for both variants.

**Figure 4:**
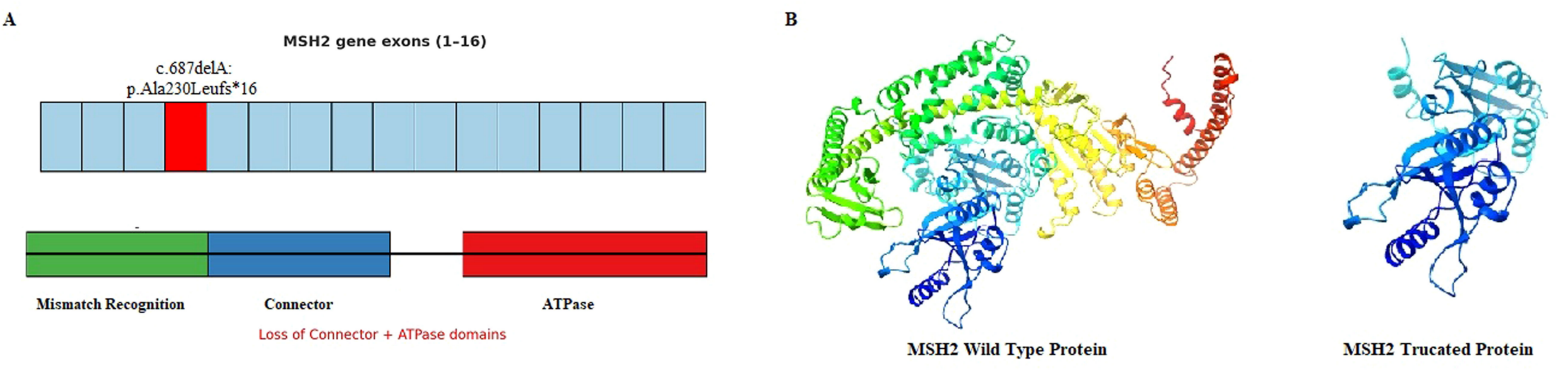
Localization of the pathogenic variant (c.687delA) in exon 4 of the *MSH2* gene and the corresponding truncated MSH2 protein **(A)**. Three-dimensional structure of MSH2 wild-type protein and the MSH2 mutant protein **(B)**.

First, targeted NGS was conducted on patient II-5, who developed CRC and carried a germline frameshift variant in exon 4 of the *MSH2* gene (NM_000251.3: c.687delA: p.Ala230Leufs16), classified as pathogenic in ClinVar and reported for the first time in Tunisian patients (**Figure 3A**). Furthermore, NGS data revealed another germline variant in exon 13 of the MUTYH gene (NM_001048174.2: c.1143_1144dup: p.Glu382Glyfs43) in patient II-5. Both variants were further verified by Sanger sequencing (**Figure 3B**). Familial co-segregation analysis for the two pathogenic variants identified by targeted NGS was conducted on the DNA of the proband (II-3), her father (I-1), her healthy sister (II-2), and her brother (II-6). We found that the proband (II-3) and her brother (II-6) carried only the MSH2 variant (c.687delA), while her father (I-1) carried both the MSH2 and MUTYH variants. The unaffected sister (II-2) was wild-type for both variants. These data are in line with the IHC and MSI results, indicating that the *MSH2* variant (c.687delA, p.Ala230Leufs*16) is the causative alteration contributing to LS in this pedigree. The frameshift variant results in a truncated MSH2 protein of 247/934 aa, lacking crucial functional domains such as the connector and ATPase domains (**Figure 4**).

However, because the proband developed ovarian cancer, we performed *BRCA1/2* genetic testing on tumor tissue. We identified a *BRCA2* pathogenic variant (NM_000059.3: c.1813delA: p.Ile605Tyrfs*9). This variant has been reported previously in public databases.

Other hereditary CRC syndromes were considered but excluded based on molecular findings.

## Discussion

In this study, we conducted genetic analysis of a Tunisian family suspected of LS and identified a pathogenic variant in *MSH2* (NM_000251.3: c.687delA) that was further found in all affected patients and absent in the unaffected individual. According to the American College of Medical Genetics and Genomics (ACMG) classification criteria, the *MSH2* mutant was classified pathogenic for LS, meeting the criteria for PVS1, PM2, and PP5. The c.687delA caused a translational frameshift with a predicted shorter MSH2 protein of 247/934 aa, losing key functional domains such as the connector and ATPase domains (12).

This pathogenic variant segregated with the disease and consequently is responsible for LS in this family. The *MSH2* variant has been reported in LS patients from various ethnic backgrounds, but, to the best of our knowledge, for the first time in Tunisian patients (13–15). We are currently expanding our study to additional Tunisian families to evaluate whether this variant may represent a recurrent driver mutation in our population.

In addition, we identified a germline *MUTYH* variant (c.1143_1144dupG) that coexists with the *MSH2* variant in two of four CRC patients in this family. Previous studies have reported this variant as recurrent in Tunisian patients diagnosed with MUTYH-Associated Polyposis (MAP) (16–18). Whether monoallelic *MUTYH* variant carriers have an increased risk of developing CRC is still highly controversial. A large meta-analysis revealed that biallelic *MUTYH* variants significantly increase the risk of CRC, while monoallelic carriers show no significant association with increased risk (19). In contrast, several studies have reported the effect of monoallelic variants in *MUTYH* on CRC development (20–22). In this study, two patients carrying both MSH2 and MUTYH variants developed CRC before age 40, similar to patients with only the MSH2 variant. This suggests that the MSH2 variant is the primary driver of disease in this family, while the monoallelic MUTYH variant likely does not significantly contribute to LS. Nevertheless, further studies are needed to clarify whether monoallelic MUTYH variants may modify disease onset or progression in affected individuals.

In the Tunisian population, germline mutations in MMR genes are responsible for at least 35% of CRC cases among patients with personal or familial histories indicative of LS (23). Previous molecular studies have reported variants mainly in *MSH2* gene such as the c.1413dupA; p.Pro472Thrfs*4 (18), c.1552C>T; p.Q518X ((24), c.2362_2363insA, p.Thr788Asnfs*11 (24, 25). In addition, Kabbage et al. reported in Tunisian patient diagnosed with diffuse GC with suspicion of Lynch Syndrome II, a *MSH2* variant (c.728G>A p.R243Q) that is involved in the MSH2-MLH1 complex stability, and thus classified as likely pathogenic (26). In our previous work, we have identified this variant in 2 unrelated LS patients, suggesting that it might be a predisposing variant for LS in a Tunisian population (27). However, analysis of a larger number of patients is required to confirm its recurrence in Tunisian patients.

It is well documented that LS is caused by germline heterozygous pathogenic variant of a single MMR gene and that 2 pathogenic variants are not usually detected in LS patients (28, 29). However, Pinto et al., reported the co-occurrence of MSH6 (p.Gln344*) and MSH2 (p. Arg929*) nonsense variants in LS patients (30). Additionally, Ferrer-Avargues et al., have identified multiple germline variants of MMR genes and other cancer-associated genes, in LS families (31). Further studies are warranted to better understand the impact of co-existing pathogenic variants in LS patients.

In conclusion, this study identifies a rare pathogenic *MSH2* variant (c.687delA) for the first time in a Tunisian family with LS, coexisting with a monoallelic MUTYH variant in some members. Our study’s strength lies in its comprehensive approach, combining clinical data, immunohistochemistry, MSI testing, targeted NGS, Sanger validation, and segregation analysis, which allowed precise identification of the causative MSH2 variant. Reporting this rare variant in a Tunisian family broadens the understanding of LS genetics in our population.

Our findings further emphasize the essential role of patient engagement in genetic counselling. Identification of the MSH2 variant not only clarified the family’s inherited cancer risk but also empowered them to make proactive decisions regarding surveillance and reproductive planning. Detection of a germline MMR pathogenic variant carries substantial clinical impact, guiding precision management strategies such as colonoscopic surveillance, systematic screening for extracolonic malignancies, and consideration of immunotherapy for MSI-H/MMR-deficient cancers. It enables cascade testing for at-risk relatives, promoting early detection and structured surveillance that can significantly reduce cancer-related morbidity and mortality in families with LS.

Our study has several limitations. First, the sample size is small, as the findings are derived from a single family. Larger cohort studies are needed to confirm the recurrence and potential population specificity of the MSH2 variant identified. In addition, the MUTYH variant was present in only two individuals, which limits our ability to draw definitive conclusions about its contribution to disease risk. Future investigations involving additional Tunisian families will be essential to determine whether this MSH2 variant represents a recurrent pathogenic alteration in this population.

## Data Availability

The original contributions presented in the study are included in the article/supplementary material, further inquiries can be directed to the corresponding author/s.
